# Brain-Adjusted Relational Leadership: A Social-Constructed Consciousness Approach to Leader-Follower Interaction

**DOI:** 10.3389/fpsyg.2021.672217

**Published:** 2021-07-13

**Authors:** Alexandros Psychogios, Nikolaos Dimitriadis

**Affiliations:** ^1^Birmingham City University, Birmingham City Business School, Birmingham, United Kingdom; ^2^University of York Europe Campus, City College, Thessaloniki, Greece

**Keywords:** relational leadership, neuroscience, leader-follower, consciousness, self-awareness, cognitive styles, social-brain theory

## Abstract

Relationship-based approaches to leadership represent one of the fastest-growing leadership fields and help us to understand better organizational leadership. Relation-based approaches emphasize the relationship and interaction between the leader and the follower. The emphasis is placed on the way that they interact and influence each other at attaining mutual goals. It is known that leaders are linked to followers and *vice versa* in a sense of responding to other's needs toward the achievement of mutual goals. Leaders and followers are an essential part of this social process implying that they are losing their traditional identity rooted in the formal organizational structure (manager-subordinate) and become inseparable actors of a co-constructing process of leadership. What is less known though is the way that leadership actors are linked to each other and in particular how they try to understand how to do that in the workplace. What is even less understood is the importance and role of consciousness in this relationship. Especially since consciousness appears to be both a fundamental and a very elusive element in human relations. Therefore, this paper conceptually explores the concept of consciousness within the context of the social brain theory to argue that leadership actors need to rethink their approach to individuality and focus on mutually dependent relations with each other. This paper contributes to the field of Neuro-management by introducing the concept of *Homo Relationalis*. In this respect, we suggest that leadership is not just a socially constructed element but also a social brain constructed phenomenon that requires an understanding of the human brain as a social organ. We further recommend a new approach of applying cognitive style analysis to capture the duality of leader/follower in the same person, following the self-illusion theory. Finally, we conclude that we need to further emphasize a *social brain-adjusted relational leadership approach* and we introduce two new cognitive styles that can help capture the essence of it.

## Introduction

Relationship-based approaches to leadership represent one of the fastest-growing leadership fields and help us understanding better organizational leadership (Dihn et al., 2014). Relation-based approaches emphasize the relationship, and thus interaction, between the leader and the follower, rather than focusing on leader or follower's characteristics and attitudes. In other words, the emphasis is placed on the way that the two human aspects of the leadership phenomenon interact and influence each other toward attaining mutual goals (Erdogan and Liden, 2002). It is known that leaders are linked to followers and *vice versa* in a sense of responding to each other's needs (Simons et al., 2011). The process of their interaction along with the result of their interaction comprise the wholeness of leadership. Viewing leadership as a relational process means that there is a mutual social influence “through which emergent coordination (i.e., evolving social order) and change (e.g., new values, attitudes, approaches, behaviors, and ideologies) are constructed and produced” (Uhl-Bien, 2006, p. 655). Leaders and followers are essential parts of this social process implying that in a relational mode of understanding leadership, they are losing their traditional identity that is rooted in the formal organizational structure (manager-subordinate) and become inseparable parts of a co-constructing process of leadership. They become, according to our view, leadership actors^1^.

What is less known is the way that leadership actors are linked to each other and in particular how they try to understand how to do that in the workplace. What is even less understood is the importance and the role of consciousness in this relationship. Especially since consciousness appears to be both a fundamental and a very elusive element in human relations. Therefore, this paper explores the concept of consciousness within the context of the social brain theory to argue that leadership actors need to revise their approach to individuality and focus on mutually dependent relations. Furthermore, we introduce the concept of *Homo Relationalis* that should replace the dominant figure of *Homo Economicus*. In this respect, we suggest that leadership is not just a socially constructed element, but also a social brain constructed phenomenon that requires an understanding of the human brain as a social organ. We further recommend a new approach of applying cognitive style analysis to capture the duality of leader/follower in the same person, following the self-illusion theory.

In order to reach the aforementioned arguments, we have employed a research approach called the Convergence Method of Evidence (CME). This method, not unlike the investigative work of detectives, strives to explain phenomena by drawing insights from diverse fields simultaneously and from multiple inquiry lines, instead of utilizing more straightforward and single-path science methods (Shermer, 2011). CME has also been called abduction, since it is neither induction nor deduction. In abduction, or inference to the best explanation, “we take the all of our background knowledge about how the word works and decide what possible explanation provides the best account of all the facts we have” (Carroll, 2016, p. 41). By combining diverse but relevant theories and research from neuroscience, evolutionary biology, anthropology, psychology, and leadership, we were able to construct a new approach in understanding leader-follower relations and to develop the neuroscience-based model we suggest.

Following the CME method, the paper is organized in six sections. In the first one, we try to review the main arguments coming from relational leadership schools and how they are associated with brain science. In the second and third sections, we explore consciousness arguing that it is linked more to a collective brain and not to an individual one. In the fourth section, we bring on the discussion social brain theory that also links relational approaches to leadership with neuroscience. In the fifth section, we show that although social consciousness and social brain theory can show us the way toward relational leadership, the evolution of leadership and leadership relations were based on a bounded view of human relations driven by the *Homo Economicous* archetype that emphasize our egoistic selves. In the last section, we summarize our main arguments and we introduce two new cognitive styles that can help capture the essence of social brain-adjusted relational leadership.

## Relational Leadership On Board

The traditional leadership theories attempted to approach and understand leadership as an individual feature that consists of many cognitive aspects. The focus was on the individual that acts as a leader and his/her traits, behaviors, and styles. In other words, leadership traditionally is viewed and explored as an isolated phenomenon that is based on the main actor, the leader, and his/her behavior formulated by his/her experience and knowledge. As Cunliffe and Eriksen (2011) argue, traditional leadership theories were based on the “*periphery* and *content* aspects of leadership” (p. 1428) not the actual sense of leadership. These approaches ignored that leadership is a social phenomenon that takes place in a social context and it is highly formulated on a continuous basis from people's actions and interactions. In this paper, we follow a different approach to leadership that is not related only to the individual level of analysis, but mainly to a dyadic and group analysis, where leadership is a part of social context full of interactions (Dionne et al., 2014). The context includes the dyadic level of analysis, but expands on the group and organizational level as well (Yammarino et al., 2005). This multilevel context of interaction includes two main leadership actors: the leader and the follower(s) (Schriesheim et al., 2001). The actors cannot be seen independently of the context that they participate as well as they cannot be seen independently of the people that relate with (Dimitriadis and Psychogios, 2020). For example a recent study found that the perceptions of leadership as well as aspiration for leadership are influenced by both cultural and socioeconomic elements (Hoyland et al., 2021). In other words, leadership occurs within the process of relating with each other, aiming in doing things in a non-static, but dynamic and continuously evolving context. In this respect, a school of thought has been developed arguing that we need to understand the nature of leadership as a relational process (Graen and Uhl-Bien, 1995; Liden et al., 1997; Murrell, 1997; Erdogan and Liden, 2002; Uhl-Bien, 2003; Dihn et al., 2014) giving birth to the *Relational Leadership Theory* (Uhl-Bien, 2006).

Relational leadership does not primarily refer to behaviors of leaders that are relationship-oriented emphasizing on compassion, support, trust, and high quality work relations (Graen and Uhl-Bien, 1995; Lipman-Blumen, 1996; Brower et al., 2000; Uhl-Bien et al., 2000). Although, we recognize the importance of these aspects in relation to consciousness and social brain, relational leadership can be understood as a social construction process within complex collective entities (organizations) and through connections and interdependences of their members (Hosking et al., 1995; Bradbury and Lichtenstein, 2000; Psychogios and Garev, 2012). The relational school argues that leadership is understood as a continuous and evolving reality within the process of organizing and occurs in interdependent relationships (Uhl-Bien, 2006). Therefore, leadership as a relational process has to be explored in the context of ongoing dynamic relations (Holmberg, 2000).

However, there are different approaches of how relational processes formulate organizational leadership realities emphasizing on dialogue and conversation, relational dynamics and creation of interactive processes (Cunliffe, 2001; Vine et al., 2008; Ness, 2009). For example, leadership is seen as a relational dialogue among organizational members, whose interaction and engagement constructs everyday organizational realities (Drath, 2001). This view of leadership is not related to a person's dominance and power of influence as traditional leadership approaches claim. Leadership is related to the way that people experience daily events and making judgments in the moment of their interactions with others in organizations about these events (Cunliffe and Eriksen, 2011; Antonacopoulou and Psychogios, 2015). With this in mind, leadership is a shared responsibility and social act rather than an individual action based on personal behaviors and characteristics (Murrell, 1997). In short, leadership is always a process of relating, and relating is a constructive, ongoing, and dynamic process of meaning making (Uhl-Bien and Ospina, 2012). In this respect, the present paper adopts a relational definition of leadership where leadership is viewed as a *never-ending meaning-making story* that is located in the ways that organizational members act and interact with each other, attempting to influence organizational understandings and produce outcomes (Barge and Fairhurst, 2008).

However, what it is less known and comprehensive from the relational approach to leadership is associated with the “how” of leadership process. It seems that there is a missing link regarding the ways that leadership is constructed as a meaning making process. We suggest that this gap can be covered if we also take into account other approaches of understanding social connection that can be found in brain science. In particular, we argue that leadership as a relational process of meaning making that is taking place through endless influential interactions in a specific context, requires the brain awareness of leadership actors (leaders and followers). In other words, relational leadership approach can be seen and understood better if we engage neuroscientific approaches.

In the next sections, we utilize on consciousness approaches and social brain theory to demonstrate our arguments. We argue that consciousness helps us to be aware of our own subjective experience of events and processes, hence relational leadership experiences as described above. This in turn, can facilitate the leadership process itself. In addition, by putting on board social brain theory, we support the view that our brains are better fit to relational experiences and therefore relational leadership as such rather than traditional (hierarchical) leadership formalities. We conclude by suggesting the need for a brain-adjusted relational approach to leadership.

## Understanding the Nature of Consciousness

Within the few scientific debates attracting major attention from media and the public imagination, consciousness holds a prominent position. Article titles such as “Why can't the world's greatest minds solve the mystery of consciousness?” in 2015 on The Guardian (Burkeman, 2015) and “World's Smartest Physicist Thinks Science Can't Crack Consciousness” in 2016 on Scientific American (Horgan, 2016) portray the levels of fascination, but also sensationalism, that the concept of consciousness attracts. Media coverage aside, understanding consciousness, and its role in human relations, might hold the key to upgrading the analysis and comprehension of relational leadership within organizations.

The main challenge of studying consciousness can be summarized in the question: can we ever fully reveal its purpose? This question has been dubbed as the Hard Problem of consciousness (Chalmers, 1995) and has been the leading conundrum for many in search of decoding consciousness. Chalmers (1995), separated the easy problems (can be solved through computational or neural mechanisms) and the hard problems (cannot be solved by computational or neural mechanisms) of studying consciousness. However, the actual hard problem of consciousness is human *experience* since “…we have no good explanation of why and how it so arises.” (Chalmers, 1995, p. 5), In addition, the soft part of this problem is about studying neural and other biological processes that are responsible for capturing stimuli, focusing our attention, controlling our behavior and integrating information cognitively or in general, various functions and abilities (Chalmers, 1995) formulating experiences. The hard part of it goes beyond function and is about subjectivity of experience: the fact that those functions could be done without being necessarily aware of them as we do, but we are.

The above approach of consciousness is a continuation of a number of philosophers' argumentation since classical antiquity and especially ancient Greece, who separated between the physical and inner worlds (Phillips et al., 2014). Talking either about *psyche* (soul) or about *nous* (mind) ancient philosophers were intrigued by subjective awareness and the fact that consciousness existed seemingly in separation from nature. These two worlds, nature and thought, body and mind, or more recently, brain and mind, has been called Dualism and it is central to the debate on consciousness (Crane and Patterson, 2012). This Cartesian substance dualism, suggests “the mind and the body as two fundamental different “things,” equally real and independent of each other…” (Grankvist et al., 2016, p. 1). In other words, according to Chalmers (1995) the dualistic approach of consciousness is about two main questions: *Why we are aware of our own subjective experience* (Hard Problem) and *How are we aware of our own subjective experiences* (Soft Problem). Although this approach does coincide with the exact nature on dualism and despite the fact that there are more dualisms (Phillips et al., 2014), Dualism itself is not universally accepted.

We argue (as many others do) that rejecting dualism might be the fastest way of solving the hard problem of consciousness focusing on the real one, which is the soft problem (Dehaene, 2014). The ‘Divide and Conquer' method of modern Dualism needs to be abandoned (Dennett, 1996) if we are to produce meaningful and useful insights of consciousness, particularly for understanding the process of leadership. This is because it is the interplay of the why and how that makes consciousness so central for the relational approach of leadership process in organizations. As Seth elegantly puts it 2016: “But there is an alternative [to the hard and soft problems], which I like to call the *real problem*: how to account for the various properties of consciousness in terms of biological mechanisms; without pretending it doesn't exist (easy problem) and without worrying too much about explaining its existence in the first place (hard problem).”

## Social Constructed Consciousness and Self Awareness

The difficulty in explaining the “Why” of consciousness might lie in the extreme importance that the western world is putting on individuality. The western notion of self has been found to be significantly more individualistic and ego-centric than in other cultures and this has a considerable impact on the subjective experience of westerners, including on their cognition and emotions (Markus and Kitayama, 1991). This acute focus on the individual and on the value of a single person as opposed to wider social units. This individualist approach, laser-focusing on the person's interests rather on communities or the multidimensional bonds within societies, is emerged from the concept of what has been called the *Homo Economicus* view of the human kind. *Homo Economicus* is an individualistic conception of humanity, void of any social dimension, which considers as natural law that the self-interest of one person is the interest of all people, leading to an ultra-egocentric model of decision making and behavior (Pesch, 2002). Actually, the *Homo Economicus* concept can be also found in traditional leadership studies, and it is consistent with a positivistic epistemology and a Cartesian dogma of a clear distinction between mind and nature (Bradbury and Lichtenstein, 2000). It assumes that individuals have a “knowing mind,” as well as that they have access to the contents of their mind (Uhl-Bien, 2006) that they can control (Hosking et al., 1995).

Dipped into neoclassical economic thinking and, most paradoxically, bound by an extreme passion for rational decision-making that always aims to maximize results and minimize costs, the *Homo Economicus* model of humanity is fading away: new models such as the *Homo Reciprocans*, the *Homo Sociologicus*, and the *Homo Socioeconomicus* have emerged as an effort to understand better the complex interrelations between people (O'Boyle, 2007). In a similar vein, we propose the term *Homo Relationalis* to show that it is not the individuals as single agents, isolated into an egoistic mind driven by rational self-interest that can help us improve our understanding of socioeconomic interaction. But it is the relational aspects (interconnectivity, interrelatedness, and interaction) between us (*Homo Relationalis*) that needs to be factored in, if we want to explain and further understand the leadership process.

Individuality, self-interest, ego-centricity, and ultra-rationality have been the guiding forces that seem to have shaped, and still shape, our approach to consciousness. If we continue to look at consciousness as a mechanism that creates subjective, thus individual, experiences then we might never understand its value and purpose. However, if we look at consciousness from a more socio-centric and relational view of humanity, taking into account the vital role of interdependency, we will probably start unraveling its true nature faster and deeper than ever before, thus solving the real problem of consciousness.

The main problem of consciousness within the *Homo Economicus* view is that since consciousness, is responsible for our subjective/individualistic experience and for our self-centered, ego-driven decision-making and behavior, then what would happen if those two exact processes were found not to be depending on conscious thinking? What, then, would consciousness be for? Our own personal survival or something else? According to Halligan and Oakley (2015) the role of consciousness seems to be linked to the function of the brain. For example, “muscles and brain areas prepare for an action, such as a reaching out for an object, before we are even aware of our intention to make that movement…consciousness simply occurs too late to affect the outcomes of the mental processes” (Halligan and Oakley, 2015, p. 26). Latest research identified the gap between the brain's unconscious preparedness for action and the conscious awareness of the action to 11 s (Koenig-Robert and Pearson, 2019). The fact that our brains prepare to take a decision much earlier than when our consciousness kicks in, brings down the self-interest foundation of the *Homo Economicus*. In addition, it gives rise to a more collective approach claiming that the sense of self comes from “our unconscious mind, and provides an evolutionary advantage that developed for the benefit of the social group, not the individual” (ibid, p. 26).

We argue that our unconscious mind broadcasts all info and decisions to our consciousness that then creates an individual construct necessary for developing strategies of adaptation in the real world. Strategies such as predicting behaviors of others, disseminating selected information and being able to adjust attitudes in relation to various on external stimuli. This means that consciousness is an emergent product of our unconscious part of the brain in order to assist us in adapting to, and interacting with, our peers in order to evolve together as a group not as individuals. We call this as a *relational approach to consciousness*. This approach captures vividly the emerging strong argument for consciousness as an evolutionary advantage (Mercier and Sperber, 2011). The importance of human communication for the species survival and growth, the ability to develop individual, conscious thoughts is for persuasion purposes and not for ego-centric decision making. Its role is inherently social.

The evolutionary advantage of a collaborative, relational, and socially adaptive consciousness has been found to hold true from other scientific disciplines as well. Evolutionary anthropology, primatology, and archeology have discovered that *homo sapiens sapiens'*, our species', unique ability to form multilayered social relations and to collaborate within highly complex and coordinated group activities with genetically unrelated individuals makes the single most important difference in species survival. Marean (2015), argues that homo sapiens' extraordinary ability to cooperate, what he has called *hyperprosociality*, which to him is not a learned tendency but a genetically encoded trait, was what helped our species dominate against other related species, such as the Neanderthals. Although cooperation can also be observed in primate species, our unique ability to collaborate in large, well-organized groups by employing a complex morality competence based on reputation and punishment was what gave the edge to humankind (De Waal, 2014). Last but not least, psychology is also revising some of its long-held beliefs on individuality and consciousness toward a more socially oriented approach. One of the most cited tests for studying self-consciousness, especially in the developmental process, is the sticker and mirror test, or otherwise called the mirror self-recognition test. In an early study Gallup (1970) measured self-awareness in non-human species and compare those to humans. Apart from humans very few other species pass the test proving that self-awareness is a function of advanced cerebral processes. Rochat (2009) conducted a similar study in non-western societies with surprising results. Instead of kids reaching for their faces, in many instances, kids were just perplexed of what they should be doing with such an unexpected situation. In particular, out of 104 kids in a Kenyan study only two removed the sticker while the rest stayed confused. Recognizing ourselves in the mirror is not about individuality, about ‘us against the world' or about finding our unique personal place in this world (Rochat, 2009). It is instead about active social engagement and formulating images of ourselves based on what others think of us. It is an outside-in test and not the other way around.

These scientific developments, pointing to a socially driven sense of self, have led many in psychology to claim that the sense of a concrete self is an illusion (Hood, 2012). In other words, our self-awareness is a fluid concept dependent on our surroundings, constantly shaped and reshaped by it. As Hohwy and Michael (2017) argue, “social interaction and cultural learning [are] key elements in the dynamic process of shaping one's self through action and interaction,” signifying the importance of the *embodied self* as a key approach in revising what it means to be us. This notion seem to be at the core of relational leadership. Many relational leadership studies argue that we are aware of ourselves as leadership actors (leaders and/or followers) based on a relational process with others. Lührmann and Eberl (2007) argue that leadership identity is co-constructed in the process of interaction between the leader and the follower. Similarly, Sluss and Ashforth (2007) claim that the role-based identities of a leader and a follower interactively “influence the [leadership] relational identity such that the [leadership] relational identity is more than the sum of its parts” (p. 13). Moreover, a follower's self-awareness is affected by leadership process itself, contemplating the effects on follower's attitudes toward leadership (Van Knippenberg et al., 2004). In other words, followers' self-conception in the leadership process is formulating in a dynamic way within the process and influenced by it. Therefore, leadership actors through the leadership process co-create a relational leadership identity.

In conclusion, adopting a more social and interpersonal view of consciousness, self-awareness, and evolution has a cascading effect on how we view our personal place in life and of course ourselves as leadership actors. First, if the human mind is not all about ourselves then consciousness is an inherent brain phenomenon that allows us to understand, relate, and interact with those around us appropriately in order to achieve various types of goals together. Second, if we are not as individuals, self-interest obsessed and ultra-rational as the *Homo Economicus* view claims that we are, then the way we set our minds to work with others should be more open, assertive and collaborative than before. Above all, our consciousness emerges unexpectedly, not as the pinnacle of human cognition and of our place in the universe, but as a product of our brains, an illusion even, that helps us create and respond to dynamic social environments and move forward more collectively as humans, professionals, leaders, and followers than individually. But in order for this to happen, we needed the right type of a brain. A “social” brain to be exact. We argue that our brain is mainly a social organ that emphasizes connecting, interacting, trusting, and cooperating, and that this is also confirmed by the evolution of human kind (Dimitriadis and Psychogios, 2020). The idea of relational leadership is based on the same foundations. We argue that as leadership actors we connect to each other in endless, dynamic, interrelated ways in various contexts that affect various outcomes. Therefore, a social brain is an essential part of relational leadership. Leadership is not just a socially constructed, but a social brain constructed process.

## Social Brain Theory and Leadership

If you ask someone for their opinion on the species that show the highest level of collaboration between their members, the typical answer that you will receive is: bees and ants. Bees' and ants' ability to collaborate within their communities harmoniously and relentlessly attracts the attention of the public. Nevertheless, the admiration of bees and ants as the ultimate cooperative machines is based on a fundamental misconception. Actually, those species do not have the decision power to choose collaboration or competition, but are directed by chemicals to collaboration (Gamble et al., 2014). In this chemically-induced “tyranny” of co-working, those species are born to cooperate with specific members of their community and for specific reasons. In an analogy to human societies, organizations would look more like totalitarian systems or like Huxley (1998) put it, like highly structured society. Actually, not counting for humans as highly collaborative species and impulsively choosing bees and ants reveals the damage done by the *Homo Economicus* mindset that focuses exclusively on competitive struggle, self-interest, and isolative individuality. Looking at the evolution of *Homo Sapiens Sapiens* in comparison with other species, and studying brain size in relation to group size, has led to the breakthrough theory of the Social Brain. This theory suggests that socializing, collaborating and co-existing in communities depends on brain size, especially frontal lobe cortical areas (Dunbar, 1998). Humans have a disproportionally big cortex/body ratio and this allows them to form larger groups with complex relations. It can be argued that, according to the Social Brain theory, higher neuro-complexity leads to higher social-complexity. Dunbar (1998) is actually famous for his optimum numbers of various social groups to have close ties between their members—which is 5, 15, 50, and 150 people depending on the closeness of the relationships-. This was found to hold true even in our highly networked era dominated by the internet and social media (Gamble et al., 2014).

The human social brain is able to behave in extraordinary ways. Based on a more complex cortex humans have the ability to reciprocate, collaborate, empathize, trust, form intelligent analysis of social situations, but also deceive and fight more cleverly than other species (Dunbar, 1998). Unfortunately, it is the latter group of those social behaviors that initially attracted many scientists who, by observing children's ability to get what they wanted from their parents, labeled these abilities as Machiavellian^2^. Again, applying a typical *Homo Economicus* mindset, people choose to see manipulation, social deception, and trickery in human children's behavior instead of social cohesion, social intelligence, and social co-existence. This narrowest of views though has been later revised to include all the socially positive behaviors creating a more realistic picture of the social brain's behavioral aspects. Interactivity, interdependence, and mutual understanding are core functions of the social brain since a very early age, leading to healthy development of the human mind as we grow and operate in complex human societies (Hood, 2012).

Two key components of the social brain theory is that first, brain processing capacity determines breadth of social relations and second, that the human kind has a unique ability to understand intentionality, in a much higher level than any other species. Concerning the first, Gamble et al. (2014) observed that cognitive load, the brain's ability to process information, is responsible for the number of people we can associate with in different social setting. Since with every new acquaintance our brain will have to process new information, and even more information for keeping regularly in touch with this new person, our brain's processing capacity will ultimately determine the ability to maintain that relation. Spunt and Lieberman (2013) have found that when cognitive load increases, our automatic mentalizing capacity, our ability to understand and connect to others, drops drastically. Thus, relationship building becomes harder. Our brain's expanded cognitive load is an actual advantage, when compared to other species, but also a limitation because of the boundaries it sets for further social bonding. Concerning the second, unlocking each others' intentionality is a building block of social interaction and since humans can manage up to six orders of intentionality, we are uniquely champions in the animal kingdom (Gamble et al., 2014).

Our intentionality-decoding skills are so advanced when compared with other species that they alone have been deemed enough to explain the Why of consciousness. Graziano (2013) suggests that we have consciousness in order to detect the consciousness of other people and thus to be able to make assumptions about their behavior. The Social Brain theory further supports the relational answer to the “Why” question (hard problem) of consciousness. The evolved human brain is set for dynamic and complex relations that are made possible through advanced intention-reading skills unique to our species. Therefore, answering the “Why” of consciousness in this manner leads to important insights into the “How” (soft problem) of consciousness too. Attention Schema Theory (AST) explains the inner-workings of awareness as an attention system that utilizes external and internal stimuli to create subjectivity, preparing the individual to act effectively to various situations (Graziano and Webb, 2015). Under this approach, consciousness is a neuro mechanism through which the brain creates mental models of reality in order to focus where needed the most. These mental models are both created by attention and result in attention. The ultimate aim is to understand other people, understand our own stance, and to respond appropriately. Although AST does not necessarily require a socially-oriented consciousness approach (Rahimian, 2021), it seems to offer an effective integration of two sister phenomena that many believe to be separate within the brain: attention and consciousness (Nani et al., 2019). In a nutshell, Graziano's AST suggests that our brains construct a simplified model of attention, leading to control of attention and creating to a conscious experience that is both internal (awareness of what is happening with us) and external (awareness of what is happening with others) (Wilterson et al., 2020).

The “Why” and the “How” are coming together, bridging the gap between purpose and function, when consciousness is viewed as an evolutionary mechanism of the embodied self that enables humans to navigate effectively and efficiently through dense and multifaceted relations within families, friendships, institutions, communities, and societies. If this is the case, then it would be logical to expect that leadership in modern organizations is guided primarily by principles of empathy, collaborating, caring, and trust. We suggest social brain-constructed relational process of leadership, it is important to understand how cognitive styles can help us rethink the leader-follower dual relationship.

## Cognitive Styles and Leadership/Followership Duality

In order to understand further the brain aspects of leadership actors, we argue that we need to take a step back, exploring through brain science the leadership-followership duality. Therefore, by focusing on consciousness, self-illusions and cognitive styles it is important to open the research agenda for further understanding of the self-dyadic relationship (leader-follower) that consist the basis of the social brain-constructed relational leadership.

The scientific debate on the evolution and function of consciousness has had intriguing side-effects on other topics, most notably on the concept of the self. The *Homo Economicus* mindset applied to consciousness as the epitome of our individuality, suggests that humans have a strong grip over their self, which they understand and control (Pesch, 2002). Most importantly, people have one, solid self, or personality, which can be captured using quantitative tools like surveys. The problem with this approach is that it does not take into account significant findings from neuroscience and other brain-related sciences pointing to a discrepancy of what we think about ourselves and what is actually happening. This discrepancy, in relation to the concept of the self, has been called the Self-Illusion. Hood (2012) explains the sense of authenticity of an essential self within us, that feels true and unified: “[t]his core self… is, however, the illusion” (p. 82).

The phenomenal experience of a subjective reality and the absence of a core self is also discussed in depth by Metzinger (2003) who suggested the Phenomenal Self-Model concept of the Self-Model Theory of Subjectivity. Metzinger (2009) claims that “[t]he phenomenal Ego is not some mysterious thing or little man inside the head but the content of an inner image- namely, the conscious self-model, or PSM. By placing the self-model within the world-model, a center is created. That center is what we experience as ourselves, the Ego” (p. 7). In other words, humans are not in direct contact with either the external nor the internal words, but they do have a representational model that feels unique and real which is much more socially-oriented and socially-derived than expected.

The point to make about the ego, or self, is that it is more of a feeling than a fixed reality. The human brain adapts its reaction in different settings and switches off and on behaviors based on genes, past experiences and social triggers. Although it feels as a continuous and consistent process, the self is an illusion and people's behaviors depend more on adjusting social brain processes than our sense of a solid self. In order to apply this approach to the relational leadership process, cognitive styles need to be discussed.

Different brains show attention and process incoming information in working memory in different ways. The speed and overall efficiency of these constitute what is called cognitive style (Happé and Frith, 2006). In organizational sciences, the concept of cognitive styles was popularized by the Cognitive Styles Index by Allinson and Hayes (1996) which proposed a questionnaire for measuring managers and employees in two variables, analysis vs. intuition, viewed as being distinct cognitive styles. Few years earlier, the Cognitive Flexibility Theory (Spiro, 1988) emerged in pedagogy, to describe efficient learning under challenging conditions. Cognitive flexibility is contrasted to cognitive rigidity when attention and perception models hinder rather than allow for learning and behavioral change (Tchanturia et al., 2004). The concept of cognitive flexibility and rigidity were popularized in the business world by the work of Dweck (2008) on growth vs. fixed mindset (Dimitriadis et al., 2018). Creative styles have also been linked to create thinking, problem-solving and innovation with the distinction of divergent creative cognitive style vs. divergent creative cognitive style (Chen et al., 2015). In leadership-related literature, the majority of work on cognitive styles has been focusing on creative organizational output and leadership (Zhang et al., 2011) rather than on leadership in general, as shown by the wider use of Kirton's adaption-innovation theory (Stum, 2009), which made the distinction between the adaptor cognitive style vs. the innovator cognitive style (Jain and Jeppe Jeppesen, 2013).

Based on the analysis of consciousness as a social tool, and the social brain and self-illusion theories, we recommend a new cognitive style distinction between the person as a leader vs. the same person as a follower (self-dyadic), with both styles being active at the same time. This means that there is a need in leadership studies to adopt a neuroscientific perspective, where the self of a person within an organization changes to fit into a leadership role, the leadership cognitive style vs. the follower role, the follower cognitive style, based on the situation. Since the presence of an authentic, one-dimensional, continuous and rigid core self has been deemed as a subjective feeling rather than a scientific reality, the change between leadership and followership cognitive styles, each with its own attentional and perceptual distinct processes applied even within the same day, but with different people and overall setting. Although still a hypothesis, such a distinction would help leadership theory progress beyond the standard view of a person as either a leader or a follower (both of them leadership actors), unlocking complex processes that might explain better the dynamic reality of multilayered relations within current organizational realities. Such a cognitively driven hypothesis fits also well with AST, which recommends three key cognitive processes present for human consciousness: endogenous control of awareness, exogenous control of awareness, and the resulting experience of a non-physical awareness of personal being (Wilterson et al., 2020).

From an academic perspective, delving into consciousness theories, social brain, and self-related theories, will help us look into the specific mechanisms of awareness, reality, and meaning creation within the context of leadership actors (leader-follower) relations and the leader/follower duality, and thus, develop further the Relational Leadership Theory (Uhl-Bien, 2006). Furthermore, our approach has the potential of contributing to the advancement of Van Vugt (2006) Evolutionary Leadership (EvoL) Theory, which more recently also includes discussions of a follower viewpoint in explaining evolutionary beneficial leadership-related behaviors (Bastardoz and Van Vugt, 2019). EvoL theory explains the way that leadership is a biological product evolved through physiological, neurological and psychological processes (Vugt, 2018). What we further suggest is that leadership as a biological product is not relational and context free, since it is highly sensitive to social relations that are occur in a specific context. In particular, our approach by combining relational and evolutional approaches to leadership suggests that leadership as a social process itself is rooted in the brains of leaders and followers that are wired and interacting in continuous, endless ways. In other words, the evolution of leadership follows an additional evolving way of interacting through our brain functions and influencing each other.

From a practice perspective, leadership actors will understand better how their own view of reality and the things they focus on influence their relations with other actors as well as how leadership actors' attention models can do the same. At the same time, leadership actors will have a better view of how their leader/follower automatic cognitive styles influence their relations and decisions. This in turn implies that the development of leadership capabilities in organizations should take into account a social brain-constructed relational approach and target the development of leadership not as a set of individual skills, but in contrast as a set of skills that are dynamic, mutually influenced, and co-created in a social context. This approach requires a good understanding of the cognitive styles of leader-follower in one person as we suggest.

## Discussion: Toward Brain-Adjusted Relational Leadership

Leadership, evolutionarily speaking, is about creating appropriate conditions and trusting relationships for group members to contribute the most they can in the group's mission. These conditions include trust, care, protection, and cohesion as necessary requirements for effective leadership (Brower et al., 2000; Uhl-Bien et al., 2000). Traditionally, the job of a leader was to provide support and safety to group members in order for them to feel liberated enough to perform their tasks in the most creative, passionate, and successful way. This community-oriented approach to leadership was found to be instrumental in how pre-historic tribes lived and survived in harsh environments. Sinek (2011) utilizing a number of sciences, such as neuroscience, evolutionary biology, and anthropology, has convincingly argued that leadership in modern companies should create circles of trust within an organization in order for its members, and the organization itself, to flourish. He claims that the negative image of big business in Western societies is exactly because of the unnatural type of leadership they apply. When leaders look only after their own interests, ignoring the welfare of their employees and the society as a whole, they do not act as our brain expects. We argue that based on the relational aspect of leadership, true leadership actors (leaders/followers) care for each other using their consciousness as a tool for the development of in-group collaboration, interdependence, and trust. These should be the key relational bonds in a continuous process of interaction among organizational members.

However, current evidence, mainly from Western societies, shows something different. The fact that business, as a societal institution, has an unfavorable image within the wider global population is well-documented. The annual Trust Barometer study by Edelman (2020), conducted in 28 countries with more than 34,000 respondents, found that increasingly, people are showing less trust to the businesses-oriented capitalistic system, with almost half of the sample (48%) claiming that the system is not working for them and the second largest group not being sure (34%). In a similar poll by Gallup (2020), only 19% of respondents in the US showed confidence in big businesses compared to 72% for the military and 75% for smaller businesses.

Corporate leadership seems to have gone exactly the opposite way of an anthropological and social brain-based leadership. *Homo Economicus* has turned leadership to a Machiavellian instrument of deceit, manipulation, and self-preservation. Essentially, leadership is turned into something that psychopaths could do uniquely well. A study has found that one out of five CEOs (formal leaders) are psychopaths (Agerholm, 2016). This number, according to the study, is equal to prison populations. This is an alarming finding, having in mind that the percentage in the total population is around 1%. Similar insights can be observed in other domains. Both the empathy deficit, the drop in overall empathy levels (Colvin, 2015), and the increase of narcissism (Twenge and Campbell, 2009) in the wider population have been much publicized (Northwestern, 2006). These intriguing facts seem to contradict the previous analysis in this paper on the nature of consciousness and the human brain as a pro-social organ. If we have been evolving to form dynamic and symbiotic relations, how is it that we are led by psychopaths and at the same time we start losing our hardwired empathic capacities? The answer is in the brain.

Our brain is plastic. New neurons are generated every day, even at a very old age, and old neurons form new connections between themselves, or severe old ones, depending on how much and how often these connections are used. These two processes, namely neuro-generation and the creation of new synapses between neurons, are fundamental to neuroplacticity (Breznitz and Hemingway, 2013). How we use our brains further enhances or weakens our mental abilities. In the case of the brain, this actually means that the software can alter the hardware, something that does not apply to manmade devices such as smartphones and laptops (Dimitriadis and Psychogios, 2020). If the requirement for managers to progress within corporations is to adopt psychopathic attitudes and behaviors, repetition will lead to permanence. Neurons that fire together, wire together (Lowel and Singer, 1992). Over time, by suppressing our empathic neural networks and boosting the narcissistic ones, we reshape our brains to mimic ones with anti-social, misanthropic and ultra-egoistic traits. The more our corporate cultures require psychopathic and narcissistic managers (leaders) the more the brain of employees (followers) will adapt to the situation. Thus, although wired around social brain, leaders and followers choose to utilize more often and with stronger efforts their non-collaborative, non-trusting, non-coexisting style resulting to brains reacting more psychopathically.

If we are to embrace the full scope of our co-operative consciousness and inter-depending social brains, we need to emphasize brain-adjusted relational leadership. A dynamic, co-created type of leadership based on relational ties of all leadership actors (leaders and followers) rather than on obsession with rigid self-interest. A type of leadership that shows a better understanding of the inner-workings and, especially, the purpose of key brain functions. A type of leadership that will bring about trust and collaboration within organizations, and that, ultimately, will unleash the true power of the *Homo Relationalis*.

Without any doubt, more conceptual and, of course, empirical studies are needed within the leadership discipline to establish the exact processes of consciousness in leader-follower relations. Future research should be open to new ways of studding leadership not abandoning the traditional socio-psychological approaches, but introducing new innovative combined methodologies. Leadership studies based on neuroscientific approaches could show the way ahead. For example, a series of leadership studies that will focus on the main biological aspects can be one category. Another category of future studies could be associated with a series of experimental research, not only from the behavioral science point of view, but also from the brain science one. Current neuro-technologies can provide huge opportunities to develop experiments and observe the actual human brain, which in turn can enhance dramatically our ability to understand human relations. For example, using electroencephalogram to measure empathy levels of managers and other professionals within a learning setting (Lambert et al., 2021). In other words, the connection of leadership with neuroscience provides endless opportunities to unlock the hidden forces that affect the way that we relate to each other and of course the way that we are involved in the leadership process. Ultimately, both leaders and followers can improve their relations and achieve more together, in a true collaborative and mutually understanding fashion. They should do this by being more confident for their socially-driven consciousness and embodied self.

## Author's Note

Both authors AP & ND contributed equally to the development of this manuscript. The process of article development was based on our search and work on the field of Neuroscience and Leadership. We first clarified the notions that we want to discuss and then start developing each sections exchanging drafts and corresponding to each other comments and suggestions.

## Author Contributions

All authors listed have made a substantial, direct and intellectual contribution to the work, and approved it for publication.

## Conflict of Interest

The authors declare that the research was conducted in the absence of any commercial or financial relationships that could be construed as a potential conflict of interest.

## Footnotes

^1^In this paper we suggest the term *leadership actors* referring to both leaders and followers that co-create and define leadership as a relational process.

^2^This characterization relates to the 16^th^ century book The Prince by Niccolo Machiavelli (2011) which has become synonymous to calculative manipulation, according to Thesaurus.com (2021).
